# Supplementary material for: Metathesis Cyclopolymerization Triggered Self-Assembly of Azobenzene-Containing Nanostructure
Source: Molecules. 2020 Aug 19;25(17):3767. doi: 10.3390/molecules25173767 (PMC7503929; doi:10.3390/molecules25173767)
Supplement: Supplementary file 1 [file molecules-25-03767-s001.pdf]

# Supplementary Materials: Metathesis Cyclopolymerization Triggered Self-Assembly of Azobenzene Containing Nanostructure

Wei Song[1,*], Jiamin Shen[1], Jinhui Huang[1], Liang Ding[1,*] and Jianhua Wu[2,*]

[1] *Department of Polymer and Composite Material, School of Materials Engineering, Yancheng Institute of Technology, Yancheng, 224051, China*

[2] *Department of Materials, College of Physics, Mechanical and Electrical Engineering, Jishou University, Jishou 416000, China*

**Correspondence: sw121092@ycit.cn (W.S.); dl1984911@ycit.edu.cn (L.D.); jianhuawu@jsu.edu.cn (J.W.);*

*Tel.: +86-0515-8829-8872 (W.S.)*

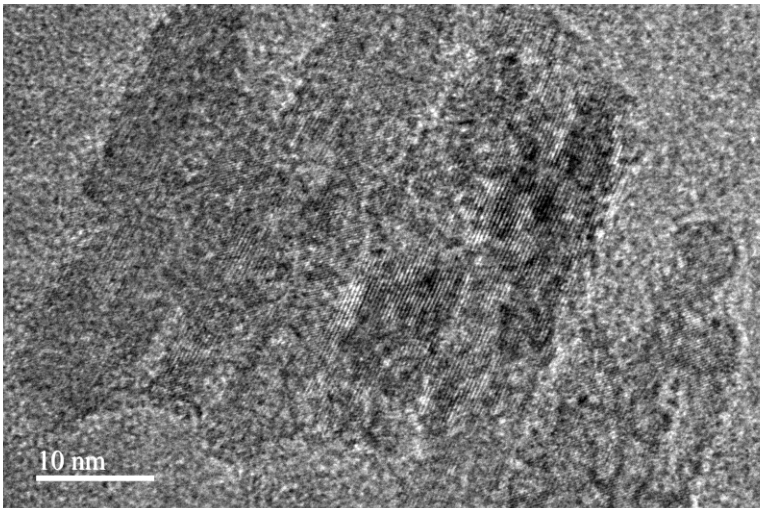


Figure S1. TEM image of double-stranded **P1**.

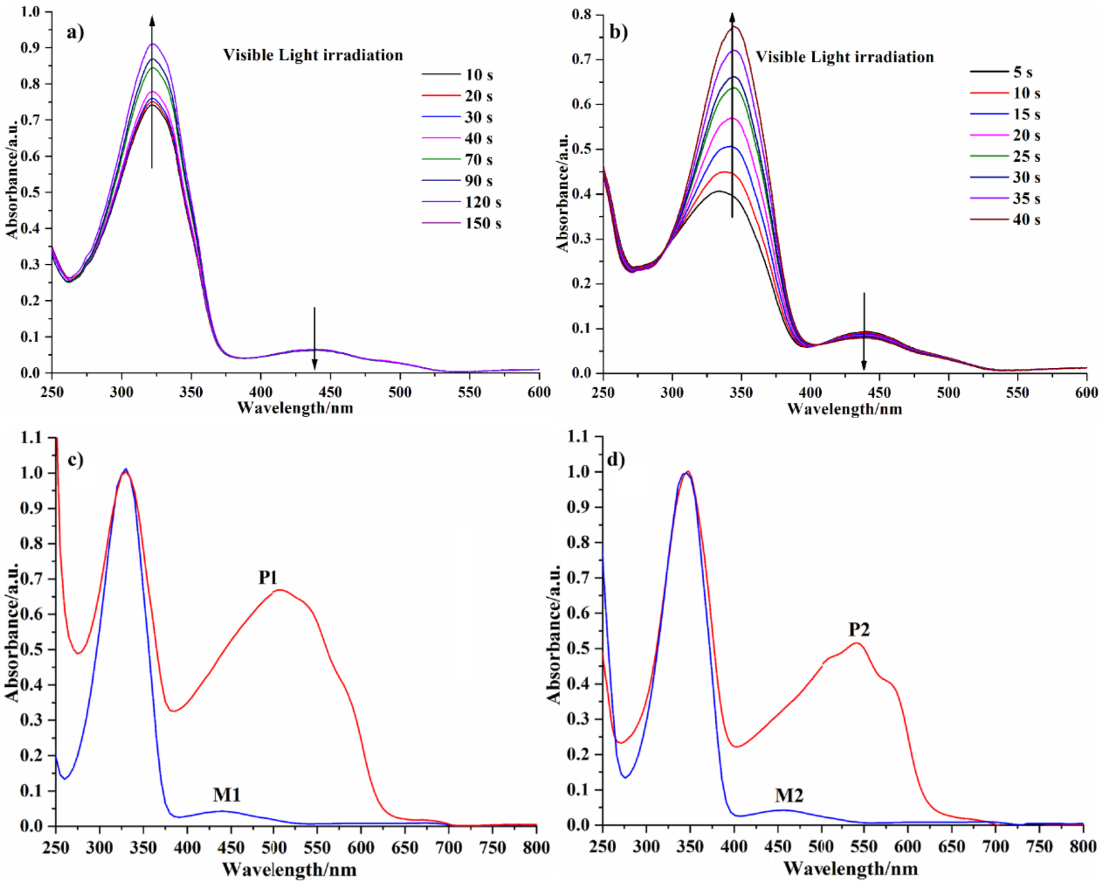


*Figure S2*. UV–Vis spectra changes for the diluted AB-incorporated a) **M1**, b) **M2**) under visible light irradiation, c) double-stranded **P1**, and d) single-stranded **P2** solutions under UV light irradiation.

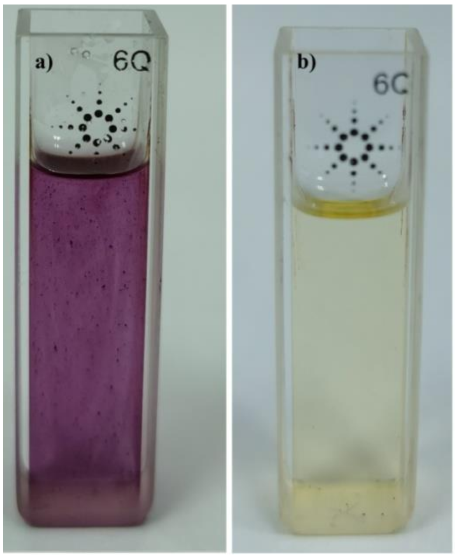


Figure S3. Pictures for conjugated polymers in THF solution: a) before UV light irradiation and b) after UV light irradiation.
